# Single-Cell Transcriptomic Profiling Uncovers a Metastasis-Associated MUCL3^+^ Signet-Ring Cell Subpopulation in Gastric Cancer

**DOI:** 10.3390/cells15100857

**Published:** 2026-05-08

**Authors:** Jie Zhang, Yinping Wang, Ting Yuan, Wenguang Wu, Xuechuan Li, Zhaoyuan Hou, Maolan Li, Yingbin Liu

**Affiliations:** 1 Department of Biliary-Pancreatic Surgery, Renji Hospital Affiliated to Shanghai Jiao Tong University School of Medicine, Shanghai 200127, China; zhangjie153000@163.com (J.Z.); wangyinping@renji.com (Y.W.); wuwenguang08@126.com (W.W.); lxclyc1234@163.com (X.L.); 2 Department of Clinical Laboratory, Renji Hospital Affiliated to Shanghai Jiao Tong University School of Medicine, Shanghai 200127, China; yuantxixi@sina.com; 3 Shanghai Key Laboratory for Tumor Microenvironment and Inflammation, Department of Biochemistry and Molecular Cellular Biology, Shanghai Jiao Tong University School of Medicine, Shanghai 200025, China; houzy@sjtu.edu.cn

**Keywords:** GSRCC, scRNA-seq, MUCL3, TFF1, metastasis-associated

## Abstract

**Highlights:**

**What are the main findings?**

- A MUCL3^+^ subcluster within the expanded Mucous_muc5ac population is spatially confirmed to correspond with classic signet-ring morphology, with MUCL3 identified as a specific protein-level marker for these cells.
- MUCL3^+^ signet-ring cells exhibit genomic instability and enrichment of TNF-α/NF-κB, TGF-β/EMT, and hypoxia pathways. Additionally, their highly expressed TFF1 is associated with gastric cancer cell migration, as validated by functional assays.

**What are the implications of the main findings?**

- MUCL3 serves as a candidate protein-level diagnostic marker for GSRCC.
- The role of TFF1 as a candidate effector warrants further investigation as a potential contributor to GSRCC progression.

**Abstract:**

**Background**: Gastric signet-ring cell carcinoma (GSRCC) is an aggressive gastric cancer subtype with abundant mucin production and high metastatic propensity. However, scarcity of specific biomarkers has impeded clinical diagnosis and mechanistic research. This study systematically compares GSRCC and gastric adenocarcinoma (AC) to identify biomarkers and elucidate molecular basis of GSRCC’s aggressive behavior. **Methods**: We performed single-cell RNA sequencing (scRNA-seq) on surgically resected primary GC tissues, validating our findings using public datasets and functional experiments. **Results**: We identified expansion of a mucin-secreting epithelial subcluster (Mucous_muc5ac) in GSRCC, characterized by high *MUC5AC*, *TFF1*, and other prognosis-associated genes. Within this population, a MUCL3^+^ subpopulation (Cluster 1) spatially corresponded with classic signet-ring morphology, validating MUCL3 as a specific marker for these cells. Multi-omics analysis revealed that MUCL3^+^ signet-ring cells exhibit genomic instability, dedifferentiation, and enrichment of TNF-α/NF-κB, TGF-β/EMT, and hypoxia pathways, with elevated metastasis/angiogenesis gene scores and high *TFF1* expression. Functional validation confirmed that TFF1 was associated with increased gastric cancer cell migration. **Conclusions**: Our study characterizes the MUCL3^+^ signet-ring cell subpopulation, highlighting the diagnostic utility of MUCL3 and suggesting TFF1 as a candidate for further investigation. These findings establish a foundation for advancing precision diagnosis and mechanistic understanding of GSRCC.

## 1. Introduction

Gastric cancer (GC), a malignancy characterized by high aggressiveness and significant heterogeneity, ranks as the fifth most common cancer globally and the fourth leading cause of cancer-related mortality worldwide [1]. In China, the annual incidence and mortality rates of GC are more than twice the global average, establishing the country as a high-risk region for this disease. According to the Lauren classification, GC is categorized into distinct histopathological subtypes, with intestinal and diffuse types being predominant. GSRCC, a prototypical diffuse-type GC, derives its name from the unique morphological feature of tumor cells containing abundant cytoplasmic mucin that displaces the nucleus to the periphery, creating a “signet ring” appearance. The World Health Organization (WHO) defines GSRCC as a GC in which signet-ring cells (characterized by prominent cytoplasmic mucin) constitute more than 50% of the tumor mass [2]. This 50% threshold defines anaplastic transformation, which also explains the therapeutic challenges associated with this subtype. GSRCC is classified as a poorly cohesive carcinoma; the neoplastic cells exhibit reduced intercellular adhesion, which facilitates early lymphatic invasion and peritoneal dissemination. Histologically, the mucosal layer in GSRCC typically exhibits a bilayer structure: the superficial layer is rich in cytoplasmic mucin, while the deeper layer contains cytoplasmic mucin and eosinophilic proteins [3]. GSRCC accounts for 35–45% of all GC cases [4], with a rising incidence in recent decades, particularly among younger female populations [5,6,7,8]. Clinically, GSRCC demonstrates aggressive biological behavior, rapid progression, and high malignancy [9,10,11]. Notably, early-stage GSRCC confined to the mucosal layer shows limited lymph node metastasis and favorable prognosis, whereas advanced GSRCC is associated with poor outcomes [12,13]. Due to low early detection rates, most GSRCC cases are diagnosed at advanced stages, where recurrence and metastasis remain primary causes of mortality [14]. Thus, identifying molecular biomarkers to accurately assess disease progression, elucidating tumorigenic mechanisms, and unraveling metastatic pathways are critical for improving clinical management.

Recent advancements in scRNA-seq have revolutionized tissue analysis, particularly in decoding tumor microenvironments, tumorigenesis, progression, and immune evasion mechanisms [15,16,17,18]. This technology enables precise characterization of gene expression profiles at single-cell resolution, uncovering cellular heterogeneity and novel subpopulations. The aggressive invasion, high metastatic propensity, and poor response to conventional chemotherapy and radiotherapy [19], along with unclear mucin secretion mechanisms and a lack of specific biomarkers, underscore the urgent need to investigate the unique biological behavior of GSRCC.

In this study, we performed scRNA-seq on paired tumor and adjacent normal tissues, systematically comparing cellular and immune microenvironmental differences between GSRCC and AC. Although Zhao et al. [20] performed comprehensive scRNA-seq on GSRCC and identified *MSMB* as a tissue-level marker, specific subpopulations within signet-ring cells remained uncharacterized. In contrast, the primary contribution of this work is the protein-level validation of MUCL3 as a specific marker of signet-ring cells, with diagnostic relevance. Secondary analyses further revealed that this subpopulation exhibits metastasis-associated molecular features and elevated *TFF1* expression, suggesting functional roles that warrant future investigation.

## 2. Materials and Methods

Single-Cell Transcriptomic Data Sources

Patients with histologically confirmed GC were recruited from Ren Ji Hospital between January 2022 and December 2023, with ethics approval (KY2021-013). None received antitumour treatments. Given the rarity of treatment-naive GSRCC, the sample size was necessarily limited. Fresh tumor and matched adjacent mucosa from three GSRCC and two AC patients (Supplementary Figure S1A) were enzymatically dissociated into single-cell suspensions. Libraries were constructed using the SeekOne^®^ DD 3’ Transcriptome Kit(K00202-08; SeekOne, Beijing, China) and sequenced on the Illumina platform (≥50,000 reads/cell). To address potential biases from the limited cohort, key findings were confirmed by independent validation (Supplementary Figure S1B) from publicly available data (GSA-Human: HRA003647).

Single-Cell Transcriptomic Data Processing

Single-cell transcriptomic data analysis was performed using Seurat (v4.1.1) (https://satijalab.org/seurat, accessed on 7 May 2026) for quality control and filtering. Cells with fewer than three detected genes were excluded. Additional filtering criteria included: UMI (Unique Molecular Identifier) counts per cell: 300–100,000; Feature (gene) counts per cell: 100–30,000; Mitochondrial gene expression Filtered using the Median Absolute Deviation (MAD) outlier detection algorithm. Batch effects were corrected via Harmony (v0.1) integration (https://github.com/immunogenomics/harmony, accessed on 7 May 2026). Following normalization, principal components were selected via the ElbowPlot method for PCA, and cellular heterogeneity was visualized using UMAP and t-SNE.

Cell Type Annotation

Cell clusters were identified using the “FindAllMarkers” function in the Seurat package, which employs a community detection algorithm to partition cells into distinct clusters. Dimensionality reduction and visualization were performed via the RunTSNE function, generating t-SNE plots to delineate cluster distributions. Major cell types were annotated based on classical marker genes and manually validated by inspecting top-ranked Differentially Expressed Genes (DEGs). For subclusters within a major cell type that lacked clear canonical markers, tentative annotation was assigned based on their top DEGs.

DEGs and Functional Enrichment Analysis

DEGs between SRCC and AC samples were identified using Seurat::FindMarkers (Wilcoxon test, min.pct = 0.1, |log_2_FC| ≥ 0.25, FDR < 0.05, Benjamini–Hochberg). These thresholds were selected to exclude low-abundance genes (min.pct), cap-ture moderate expression changes (|log_2_FC|), and control for multiple testing (FDR). Over-representation analysis (ORA) for Hallmark gene sets and KEGG pathways was performed with the clusterProfiler R package using Fisher’s exact test (FDR < 0.05, BH).

For each sub-cluster, all statistically significant marker genes (FDR < 0.05, BH, |log_2_FC| ≥ 0.25) were uploaded to Metascape (http://metascape.org, accessed on 7 May 2026) with default parameters (*p* value < 0.01, FDR < 0.05, min overlap = 3) to obtain Hallmark enrichment plots, as described in Zhou et al., Nature Communications 2019, 10:1523 [21].

InferCNV

The R package InferCNV enables the identification of copy number variations (CNVs) in tumor samples. It detects chromosomal copy number alterations, including amplifications or deletions, in malignant cells by comparing gene expression profiles against normal reference cells, thus facilitating the characterization of tumor-specific genomic features.

CytoTRACE Analysis

To infer the developmental potential of single cells, we employed CytoTRACE, a computational framework that predicts cellular differentiation states based on the diversity of gene expression profiles. This unsupervised method calculates gene count signatures for individual cells, optimizes scoring metrics, and integrates validation through dimensionality reduction visualization and established biomarkers. CytoTRACE provides a robust approach for identifying progenitor cells and reconstructing developmental hierarchies within heterogeneous single-cell datasets, offering critical insights into the dynamics of cellular differentiation.

Pseudotime analysis

Pseudotime analysis was performed using Monocle2 to reconstruct the differentiation trajectory among the four subclusters of Mucous_muc5ac. Genes with mean expression ≥ 0.1 and empirical dispersion ≥ 1 were selected for trajectory reconstruction. Dimensionality reduction was performed using the DDRTree algorithm, and cells were ordered along the pseudotime axis based on transcriptomic similarity. The root of the trajectory was manually set to the subcluster with the highest CytoTRACE score.

Tissue Specimens

Paraffin-embedded tumor tissues and paired adjacent normal tissues from 10 gastric cancer patients (7 GSRCC and 3 AC) were acquired from Renji Hospital, Shanghai Jiao Tong University School of Medicine. All histological sections were independently validated by two board-certified pathologists. This study was approved by the Institutional Review Board of Renji Hospital (Ethics Approval No.: KY2021-013) with written informed consent from all participants.

Hematoxylin–Eosin (HE)

Following deparaffinization and dehydration, tissue sections were rinsed with distilled water and subjected to HE staining using a commercial kit (C8073-1; TITAN, Shanghai, China). The staining protocol initiated with 10-min hematoxylin immersion for nuclear staining, followed by 10-s tap water rinsing and 5-min bluing in tap water to optimize nuclear contrast. Subsequent cytoplasmic counterstaining involved sequential treatment with 95% ethanol (1 min), eosin Y solution (15–30 s), and 75–85% ethanol wash (30 s). Dehydration was achieved through graded ethanol series: 95% ethanol (2 min), 95% ethanol (5 min), followed by two 5-min absolute ethanol immersions. Tissue transparency was enhanced via two 1-min xylene incubations, culminating in permanent mounting with neutral resin. Stained sections were visualized and imaged under bright-field illumination using an inverted microscope.

Immunofluorescence (IF)

Paraffin-embedded gastric tissues from signet-ring cell carcinoma patients were sectioned at 4 μm thickness. For immunofluorescence, sections underwent deparaffinization in xylene and rehydration through graded ethanol series. Antigen retrieval was performed using pre-heated sodium citrate buffer (pH 6.0). After quenching endogenous peroxidase with 3% H_2_O_2_ and blocking with 5% bovine serum, sections were incubated overnight at 4 °C with primary antibodies against MUC5AC (Rabbit, 1:200, 30408-1-AP; Proteintech, Rosemont, IL, USA), MUCL3 (Rabbit, 1:200, NBAB-109557; Nebulabio, Beijing, China), TFF1 (Rabbit, 1:200, 13734-1-AP; Proteintech, Rosemont, IL, USA), and TFF2 (Rabbit, 1:200, 13681-1-AP; Proteintech, Rosemont, IL, USA). Following PBS washes, fluorophore-conjugated secondary antibodies were applied for 1 h at room temperature. Nuclei were counterstained with DAPI, and slides were mounted in 75% glycerol for imaging under an inverted fluorescence microscope.

Multiplex immunofluorescence (mIF)

Signal amplification was performed by Hunan Aifang Biotechnology Co., Ltd. under our supervision. The staining protocol followed standard TSA procedures involving sequential labeling with primary antibodies (MUCL3, TFF1, MUC5AC), HRP-conjugated secondaries, and fluorescent tyramides (TYR-650, TYR-570, TYR-520), with heat-mediated antibody stripping between rounds. All stained sections were validated by our team prior to imaging on a multi-channel fluorescence slide scanner.

Quantitative assessment

Quantitative assessment was performed on multiplex mIF images. Signet-ring cells were identified by crescent-shaped TFF1/MUC5AC staining with eccentric DAPI nucleus. Three MUCL3-positive GSRCC samples were analyzed. For each sample, three high-power fields (40× objective) were pooled and counted by two blinded observers. The proportion of triple-positive cells (MUC5AC^+^/MUCL3^+^/TFF1^+^) among signet-ring cells was calculated per sample; median and range across samples are reported.

RNA Extraction, Reverse Transcription, and qPCR

Total RNA was isolated from 6 pairs of gastric tumor and adjacent non-tumor tissues (3 pairs of GSRCC and 3 pairs of AC) using TRIzol (15596026; Invitrogen, Carlsbad, CA, USA) according to the manufacturer’s instructions, and quantified by NanoDrop (OD260/280 1.8–2.2). Two micrograms of RNA were reverse-transcribed with SuperScript™ II Reverse Transcriptase (18064-014, Invitrogen, Carlsbad, CA, USA) in a 20 µL reaction volume. qPCR was performed on a LightCycler 480 (Roche, Rotkreuz, Switzerland) using *TFF1* primers (F: 5′-GAGAACAAGGTGATCTGCGC-3′; R: 5′-TGGTATTAGGATAGAAGCACC-3′) and *GAPDH* as endogenous control (primer efficiency 90–110%, R^2^ ≥ 0.99). Thermal cycling conditions were: 95 °C for 30 s, followed by 40 cycles of 95 °C for 5 s and 60 °C for 30 s, with a melting curve analysis at the end. Due to limited tissue yield, each sample was run in technical duplicate; the mean Ct value was used for downstream calculation. *TFF1* expression was calculated using the 2^−ΔCt^ method with *GAPDH* as reference. Data are presented as mean ± SEM from *n* = 3 biologically independent samples per group. Groups were compared using unpaired two-tailed Student’s *t*-test (GraphPad Prism 8); due to the limited sample size, the *t*-test was applied for exploratory purposes. *p* < 0.05 was considered statistically significant (* *p* < 0.05, ** *p* < 0.01, *** *p* < 0.001).

Cell Culture

The MGC803 and MKN45 cell lines were kindly provided by Dr. Zhaoyuan Hou’s laboratory at Shanghai Jiao Tong University School of Medicine. Cells were maintained in RPMI-1640 medium (Gibco) supplemented with 10% fetal bovine serum (FBS; Gibco) and 1% penicillin-streptomycin (Gibco) at 37 °C in a 5% CO_2_ humidified incubator, and were passaged using trypsin-EDTA upon reaching 80–90% confluence. These cell lines are well-characterized gastric cancer models widely used in functional studies, enabling comparison with existing TFF1 literature.

Transwell Migration Assay

Cells in the logarithmic phase were trypsinized, washed, and resuspended in serum-free DMEM at 1 × 10^5^–1 × 10^6^ cells·mL^−1^. The lower chambers received 600 μL of DMEM with 10% FBS, supplemented with or without TFF1 protein for experimental or control groups, respectively. Then, 200 μL of cell suspension was added to each upper chamber (*n* = 3 wells/group). After 24 h incubation at 37 °C, non-migrated cells were removed from the upper membrane surface. Migrated cells on the lower surface were fixed with 4% paraformaldehyde, stained with 0.1% crystal violet, and counted under a microscope. No matrigel coating was used.

## 3. Results

This identification of major cell types in tumor and adjacent non-tumor tissues

**Figure 1:**
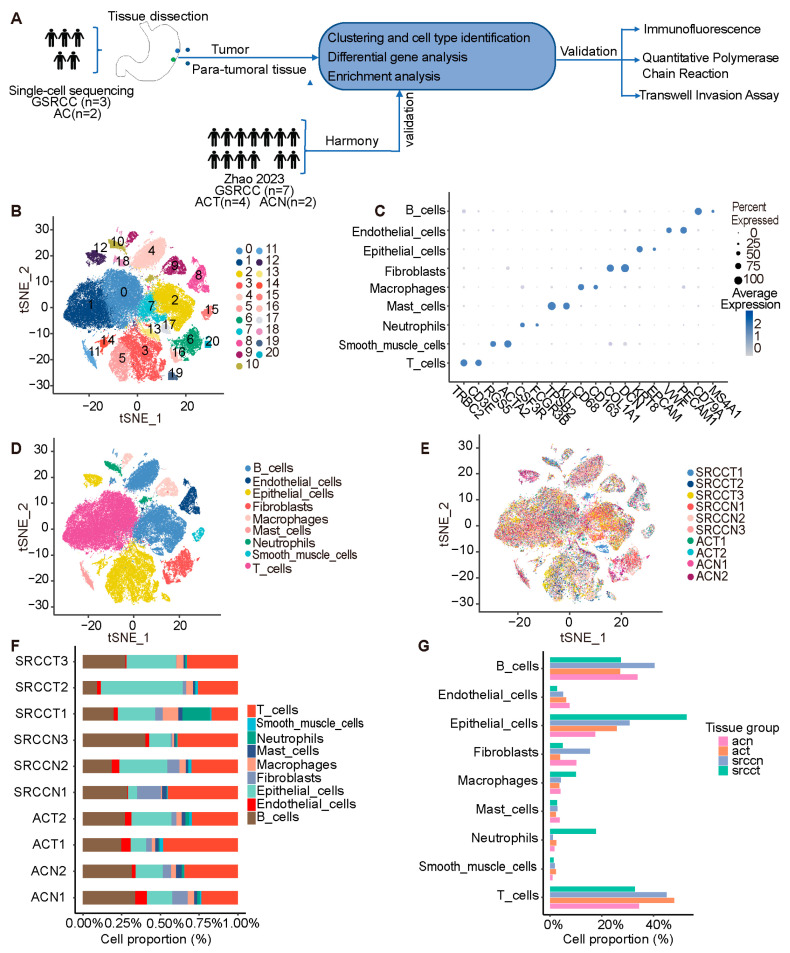
Identification of major cell types in tumor and adjacent non-tumor tissues. (**A**) Schematic overview of the study cohorts. The discovery cohort included matched tumor and adjacent non-tumor tissues (*n* = 5 pairs) from 3 GSRCC and 2 AC patients. The validation cohort comprised public single-cell data from 7 GSRCC, 4 AC, and 2 adjacent non-tumor samples. Key findings were validated using IHC and qPCR. (**B**) tSNE plot displaying 83,597 quality-controlled cells, partitioned into 21 distinct clusters. (**C**) Dot plot illustrating the expression levels of canonical marker genes across the 9 identified cell types. (**D**) t-SNE visualization colored by the annotated cell types. (**E**) t-SNE plot colored by sample origin. (**F**) Stacked bar chart showing the proportional distribution of cell types across individual patient samples. (**G**) Bar graph comparing the average composition of major cell populations across different tissue types.

scRNA-seq profiling of 10 tissue samples (5 GC patients) established a discovery cohort of 83,597 high-quality cells, with the key findings validated in an independent cohort (*n* = 13) (Figure 1A). Following data dimension reduction, we obtained 21 cell clusters (Figure 1B). Cell types were annotated based on previous literature reports [22,23,24] and known markers, where *TRBC2*, *CD3E* marked T cells, *CD79A*, *MS4A1* marked B cells, *ACTA2*, *RGS5* marked smooth muscle cells, *SDC1*, *MZB1* marked plasma cells, *KRT18*, *EPCAM* marked epithelial cells, *CSF3R*, *FCGR3B* marked neutrophils [25,26,27], *KIT1*, *TPSAB2* marked mast cells, *CD68*, *CD163* marked macrophages, *VWF*, *PECAM1* marked endothelial cells, and *COL1A1*, *DCN* marked fibroblasts. Dot plot analysis confirmed the annotation accuracy of the nine major cell types identified (Figure 1C). t-SNE visualization illustrated the distribution of the annotated cell types (Figure 1D), while analysis of cellular composition across samples (Figure 1E) revealed a uniform cell distribution across patients, indicating successful batch correction. Quantitative assessment of cell proportions revealed pronounced compositional heterogeneity across individual samples and tissue origins (Figure 1F,G). The significant heterogeneity in cellular composition according to tissue origin was reproducible in an independent validation cohort, which showed highly consistent cellular proportion distributions (Supplementary Figure S2A–E). These findings, consistent across cohorts, underscore the deterministic role of the tissue microenvironment in shaping cellular lineages and provide a statistical foundation for subsequent biological inferences.

Analysis of the major epithelial cell subpopulations

**Figure 2:**
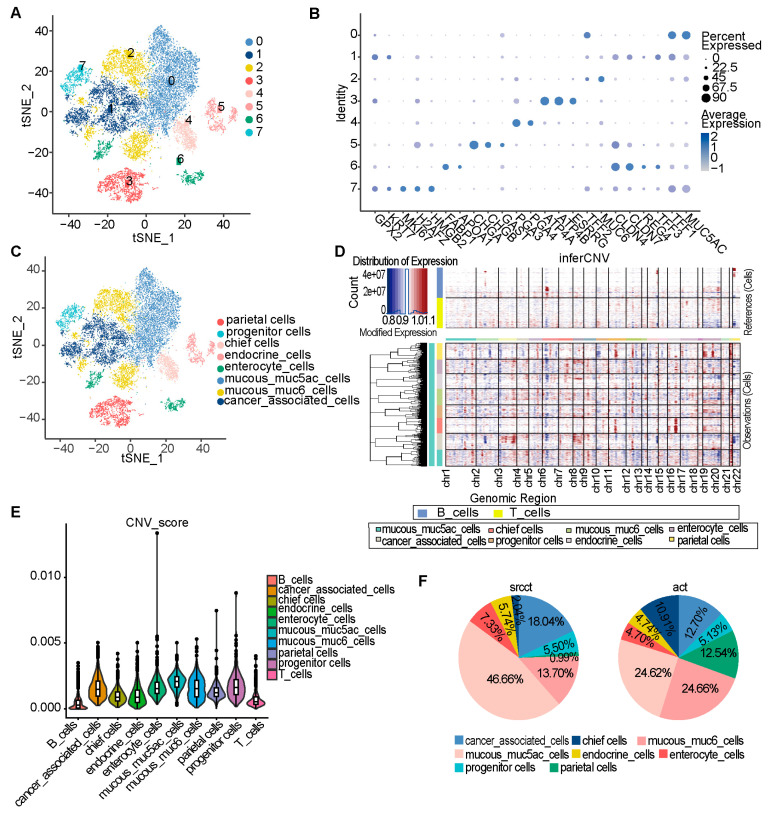
Identification of major epithelial cell subpopulations. (**A**) t-SNE visualization of 18,085 high-quality epithelial cells, partitioned into 8 clusters. (**B**) Dot plot showing the expression of canonical marker genes for the 8 epithelial subpopulations. (**C**) t-SNE plot colored by epithelial cell subtype annotation. (**D**) Heatmap of inferCNV-predicted copy number variations (CNVs) across epithelial subpopulations (red, amplification; blue, deletion). (**E**) Distribution of CNV scores for each epithelial subpopulation. (**F**) Pie chart showing the proportional distribution of epithelial subpopulations in GSRCC and AC samples.

Given the unique mucus-rich phenotype of GSRCC, this study focused on epithelial cells. After re-dimensionalization and clustering of epithelial cells from all samples (Figure 2A), eight subpopulations were identified based on characteristic genes reported in literature [20,28,29] (Figure 2B,C). Cancer-associated cells were defined by markers *CLDN4*, *CLDN7*, *REG4*, *KLK10*, *GPX2*, and *KRT7*; progenitor cells by *MKI67*, *H2AFZ*, and *HMGB2*; endocrine cells by *CHGA*, *CHGB*, and *GAST*; parietal cells by *ATP4A*, *ATP4B*, and *ESRRG*; chief cells by *PGA3* and *PGA4*; and intestinal epithelial cells by *FABP1* and *APOA1*. Based on the high expression of gastric pit mucous cell markers *TFF1* and *MUC5AC*, subcluster 0 was defined as the Mucous_muc5ac subpopulation. Similarly, subcluster 2, which expresses *MUC6* and *TFF2*, was designated as Mucous_muc6 subpopulation. CNV analysis revealed increased copy number variations in cancer-associated cells, progenitor cells, and intestinal epithelial cells compared to chief cells, parietal cells, and endocrine cells, consistent with previous studies [20]. It is noteworthy that mucous cells (particularly the Mucous_muc5ac subpopulation) demonstrated comparatively high copy number variation (CNV) levels (Figure 2D,E). Moreover, a significant increase in the proportion of these cells was found in GSRCC relative to AC upon comparison of epithelial subpopulations (Figure 2F). This pattern was consistently replicated in the validation cohort (Supplementary Figure S3A–C).

DEGs analysis between GSRCC and AC epithelial cells

**Figure 3:**
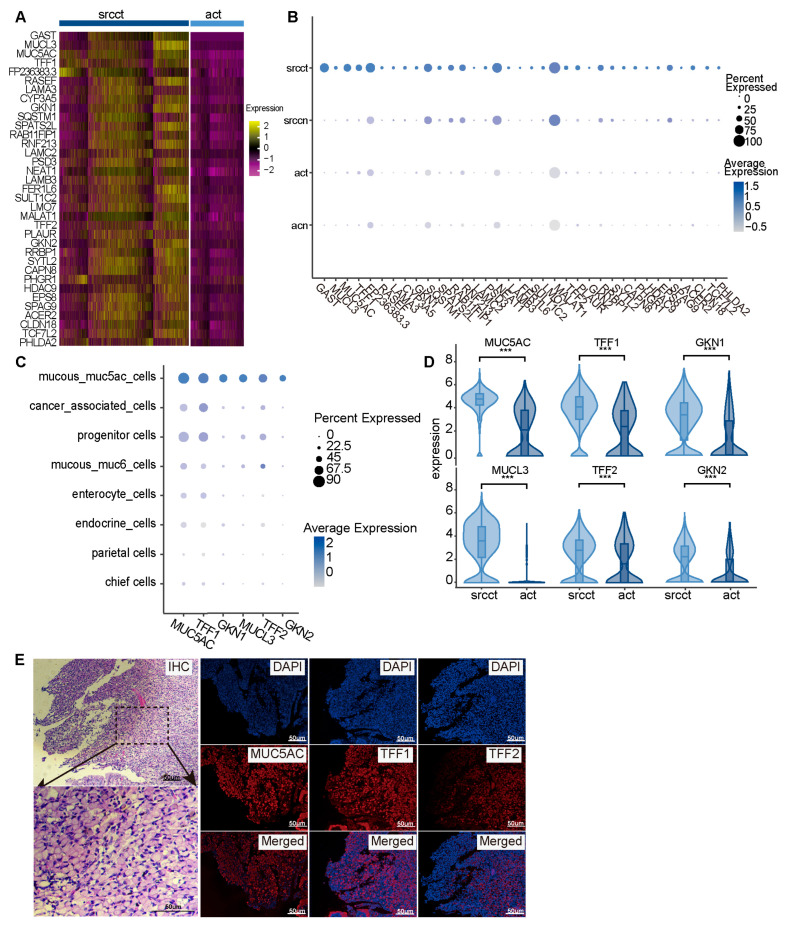
DEGs analysis between GSRCC and AC epithelial cells. (**A**) Heatmap of the top 35 genes significantly upregulated in GSRCC versus AC epithelium (Wilcoxon rank-sum test, *p* < 0.05). (**B**) Dot plot showing the expression patterns of the top 35 genes in epithelial cells across different tissue types. (**C**) Dot plot showing the expression of marker genes associated with gastric mucus and digestive enzyme secretion across epithelial subpopulations. (**D**) Violin plots comparing the expression of gastric mucus and digestive enzyme-related marker genes in the Mucous_muc5ac subpopulation between GSRCC and AC (Wilcoxon rank-sum test, *** *p* < 0.001). (**E**) Immunofluorescence staining confirms the expression of MUC5AC, TFF1, and TFF2 in signet-ring cells. The first section was stained with H&E to confirm typical signet-ring cell morphology. The remaining three sections were stained for MUC5AC, TFF1, and TFF2, respectively. For each marker, images show DAPI-stained nuclei (blue), antibody staining (red), and merged view. Scale bar: 50 µm.

GSRCC is distinct from AC in its cellular morphology, biological behavior, tissue origin, and molecular expression profile. To systematically identify characteristic molecular markers of GSRCC, we conducted transcriptomic differential analysis on epithelial tissues from GSRCC and AC, identifying the top 35 genes significantly upregulated in GSRCC (Figure 3A). These genes exhibited a highly specific and consistent upregulation pattern in GSRCC (Figure 3B). Among them, consistent upregulation of *GAST*, *MUCL3*, *MUC5AC*, *TFF1*, *RASEF*, *LAMA3*, *GKN1*, *SQSTM1*, *SPATS2L*, *RAB11FIP1*, *LAMC2*, *PSD3*, *NEAT1*, *LAMB3*, *FER1L6*, *SULT1C2*, *LMO7*, *TFF2*, *PLAUR*, *GKN2*, *SYTL2*, *CAPN8*, *HDAC9*, *EPS8*, *CLDN18*, *TCF7L2*, and *PHLDA2* was confirmed in an independent validation cohort (Supplementary Figure S4A). Further analysis showed that genes including *GAST*, *RASEF*, *LAMA3*, *SQSTM1*, *SPATS2L*, *LAMC2*, *PSD3*, *NEAT1*, *LAMB3*, *LMO7*, *SYTL2*, *HDAC9*, *TCF7L2*, and *PHLDA2* were not only upregulated in GSRCC epithelial tissues but also showed a significant positive correlation with poor prognosis in GC patients (Supplementary Figure S4B), suggesting their potential as prognostic biomarkers for GSRCC. Notably, among the top 35 up-regulated DEGs in GSRCC, several involved in gastric mucus and digestive enzyme function—including *MUCL3*, *MUC5AC*, *TFF1*, *TFF2*, *GKN1*, and *GKN2*—were significantly and specifically enriched in the Mucous_muc5ac subpopulation (Figure 3C,D). Importantly, immunofluorescence staining revealed, for the first time, high expression of MUC5AC, TFF1, and TFF2 in signet-ring cells (Figure 3E). This finding not only validates the transcriptomic results but also precisely localizes the overexpression of these molecules to signet-ring cells, providing the first protein-level evidence of TFF1 and TFF2 expression in these cells and offering insight into their potential cellular origin.

Molecular and biological study of mucous_muc5ac subpopulation in GSRCC and AC

**Figure 4:**
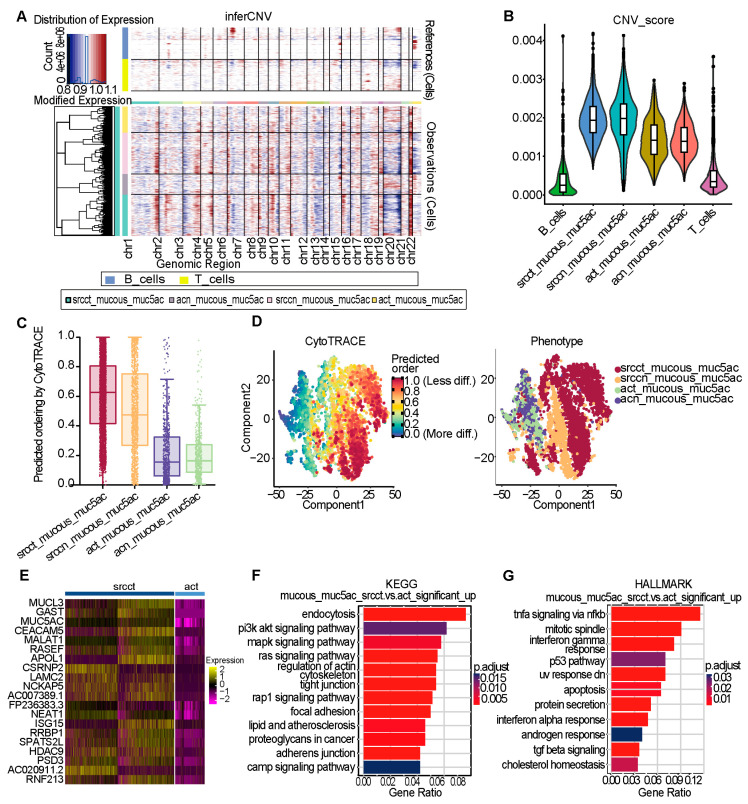
Molecular and Biological Study of Mucous_muc5ac Subpopulation in GSRCC and AC. (**A**) InferCNV heatmap of the Mucous_muc5ac subpopulation across different tissue types. (**B**) Violin plots showing the distribution of CNV scores in Mucous_muc5ac subpopulation of different tissue types. (**C**) Box plot illustrating the differentiation potential (CytoTRACE score) of the Mucous_muc5ac subpopulation from different tissue types. (**D**) Scatter plot projecting CytoTRACE scores onto the t-SNE embedding of the Mucous_muc5ac subpopulation from different tissue types. (**E**) Heatmap of the top 20 significantly upregulated DEGs in GSRCC-derived versus AC-derived Mucous_muc5ac subpopulations (Wilcoxon rank-sum test, *p* < 0.05). (**F**) KEGG pathway enrichment analysis of upregulated DEGs in GSRCC versus AC Mucous_muc5ac subpopulation. (**G**) HALLMARK pathway enrichment analysis of upregulated DEGs in GSRCC versus AC Mucous_muc5ac subpopulation.

Based on previous systematic analyses of epithelial cells in GSRCC and AC, we identified the unique biological role of the Mucous_muc5ac subpopulation in GSRCC. The significant enrichment of this subpopulation is closely associated with the characteristic mucus-secreting phenotype of GSRCC, suggesting its potential critical role in tumorigenesis and progression. Characterization of the Mucous_muc5ac subpopulation using inferCNV revealed significant CNV accumulation in its GSRCC-derived subset (Figure 4A,B). Further evaluation of differentiation potential via the CytoTRACE algorithm revealed strong dedifferentiation characteristics in the Mucous_muc5ac subpopulation of GSRCC (Figure 4C,D). Of note, CytoTRACE scores reflect transcriptomic diversity as a proxy for differentiation state rather than a direct measure. Molecular profiling revealed significant transcriptomic remodeling in the Mucous_muc5ac subpopulation of SRCC epithelial cells compared with those of AC (Figure 4E), Among the top 20 upregulated DEGs in this subpopulation, both mucin-related factors (e.g., *MUCL3*, *MUC5AC*) and other genes (e.g., *GAST*, *CEACAM5*, *NEAT1*) were specifically associated with poor patient prognosis (Supplementary Figures S4B and S5A), further highlighting the clinical relevance of these molecular markers. Pathway analysis (Figure 4F,G) revealed that up-regulated DEGs in GSRCC were enriched in TNF-α/NF-κB signaling, PI3K-Akt pathway, and mucin-type mucus production (KEGG/HALLMARK, adj. *p* < 0.05). The consistent enrichment of TNF-α, NF-κB, and TGF-β pathways aligns with established signet-ring cell functions [20], confirming their pivotal roles in GSRCC.

Single-cell transcriptomic analysis of Mucous_muc5ac subclusters.

**Figure 5:**
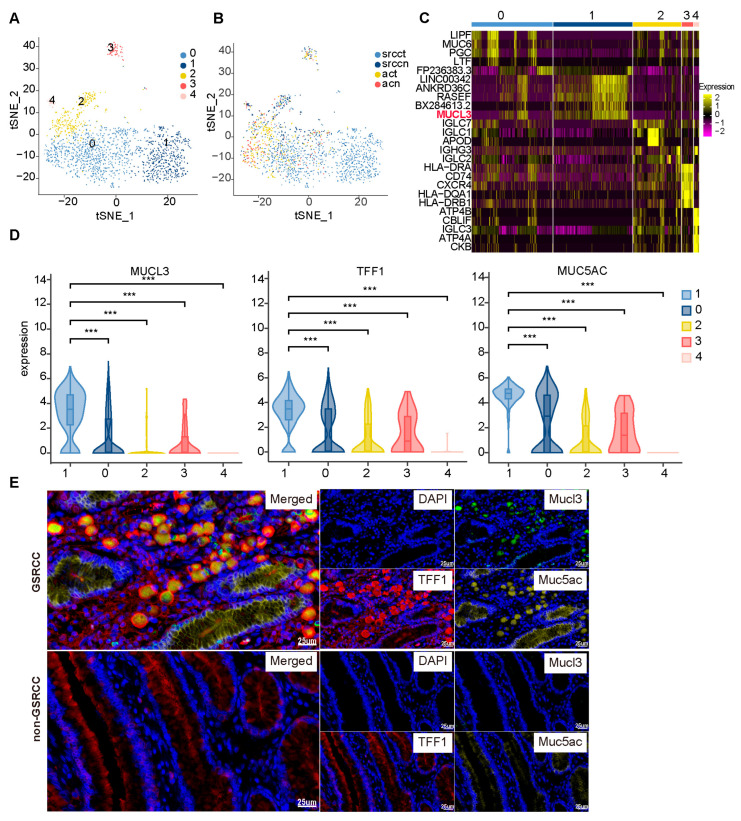
Single-cell transcriptomic analysis of Mucous_muc5ac subclusters. (**A**) t-SNE plot of the Mucous_muc5ac subpopulation, colored by subcluster. (**B**) t-SNE plot of the Mucous_muc5ac subpopulation, colored by tissue origin. (**C**) Heatmap of the top 5 marker genes for each Mucous_muc5ac subcluster. (**D**) Violin plots showing the expression levels of *MUC5AC*, *MUCL3*, and *TFF1* across Mucous_muc5ac subclusters (Wilcoxon rank-sum test, *** *p* < 0.001). (**E**) Immunofluorescence images showing the expression and localization of MUC5AC, MUCL3, and TFF1 in representative GSRCC and non-GSRCC tissue sections. Triple-positive cells (MUC5AC^+^/MUCL3^+^/TFF1^+^) exhibited classic signet-ring morphology in a subset of GSRCC samples. Non-signet-ring tumor cells showed no MUCL3 expression. Scale bar: 25 µm.

To delineate cellular heterogeneity within the Mucous_muc5ac subpopulation, we performed unsupervised clustering of genome-wide expression profiles, which identified five distinct subclusters (Clusters 0–4; Figure 5A). Of these, Cluster 1 was uniquely and significantly enriched in GSRCC tissue specimens (Figure 5B). A per-patient breakdown of cell numbers within each Mucous_muc5ac subcluster is provided in Supplementary Table S1. Molecular characterization revealed that this subcluster was defined by high and specific expression of the mucin gene *MUCL3* (Figure 5C), alongside coordinated upregulation of established signet-ring cell markers *MUC5AC* and *TFF1* (Figure 5D). These findings were validated in an independent cohort, which confirmed the elevated expression of *MUCL3* and mucus-digestive enzyme genes in GSRCC compared to AC (Supplementary Figure S6A,B). Furthermore, re-clustering of the Mucous_muc5ac subpopulation demonstrated that high *MUCL3* expression was selective to the GSRCC-associated cells (Supplementary Figure S6C–E). Based on its distinct spatial enrichment and molecular signature, we hypothesized that this subcluster represents a characteristic signet-ring cell population with diagnostic relevance for GSRCC. To test this, we performed multiplex immunofluorescence on GSRCC and non-GSRCC tissue sections. Strikingly, cells that were triple-positive for MUC5AC, MUCL3, and TFF1 exhibited classic signet-ring morphology (Figure 5E), thereby providing the first protein-level evidence for specific MUCL3 overexpression in signet-ring cells. MUCL3 expression in signet-ring cells was heterogeneous across GSRCC samples, with some cases showing triple-positive cells and others showing only MUC5AC^+^/TFF1^+^ without MUCL3, confirming that MUCL3 marks a specific subpopulation. In MUCL3-positive cases, approximately 73% of signet-ring cells were triple-positive (median 73.0%, range 70.8–77.8%), indicating that within positive samples, MUCL3 marks a major but not universal subpopulation of gastric-type signet-ring cells.

Molecular and functional characterization of the MUCL3^+^ subpopulation in GSRCC.

**Figure 6:**
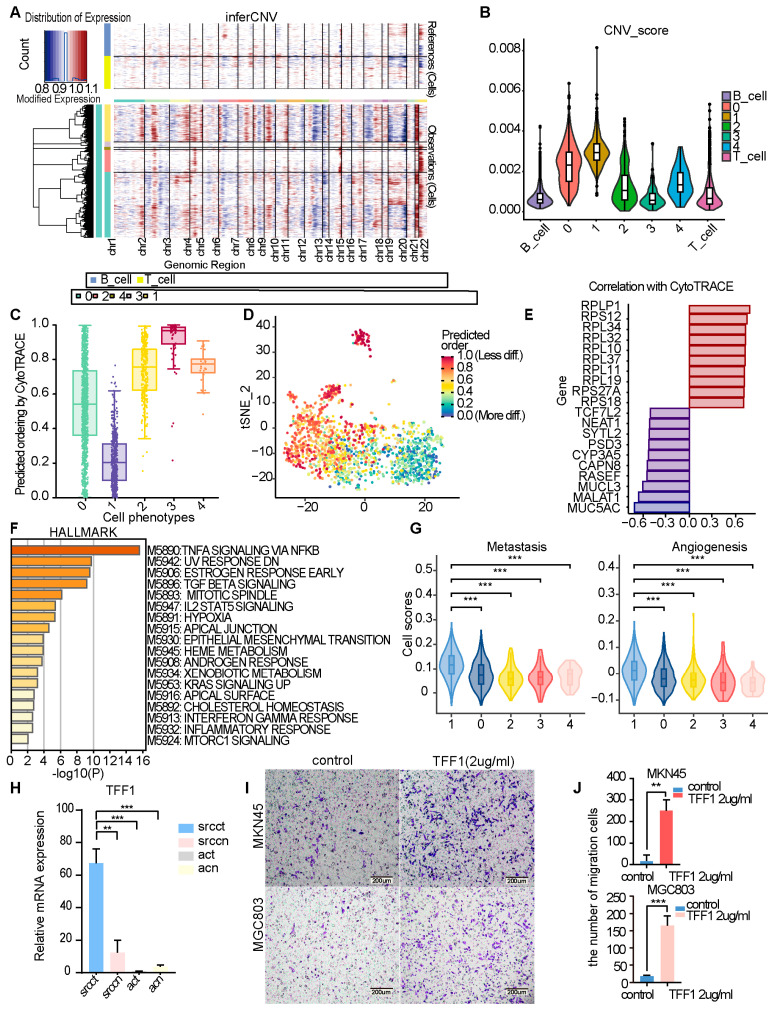
Molecular and functional characterization of the MUCL3^+^ subpopulation in GSRCC. (**A**) Heatmap of inferCNV-predicted CNVs across Mucous_muc5ac subclusters. (**B**) Violin plots showing the distribution of CNV scores for each Mucous_muc5ac subcluster. (**C**) Box plot visualizing the differentiation potential (CytoTRACE score) of Mucous_muc5ac subclusters. (**D**) CytoTRACE score projection mapped onto different cell types in the t-SNE embedding. (**E**) Top 10 genes showing the most significant positive (blue) and negative (pink) correlations with CytoTRACE scores. (**F**) HALLMARK pathway enrichment analysis of genes highly expressed in the MUCL3^+^ subcluster. Bars represent –log_10_(*p* value); the dashed line indicates *p* = 0.01. Analysis was performed using Metascape. (**G**) Violin plots displaying module scores for Metastasis and Angiogenesis in each Mucous_muc5ac subcluster (Student’s *t*-test, *** *p* < 0.001). (**H**) qRT-PCR analysis of *TFF1* mRNA expression in GSRCC and AC tissues (*n* = 3 per group). *TFF1* expression was normalized to *GAPDH*. Data are mean ± SEM. Student’s *t*-test; ** *p* < 0.01, *** *p* < 0.001. (**I**) Representative images showing the migration of MKN45 and MGC803 cells treated without (left) or with (right) TFF1 protein (2 μg·mL^−1^) for 24 h. Scale bar: 200 µm. (**J**) Quantitative analysis of migrated cell numbers from (**I**) (Student’s *t*-test, ** *p* < 0.01, *** *p* < 0.001).

Integrated multi-omics analysis revealed that the MUCL3^+^ signet-ring cell subpopulation exhibits highly malignant molecular features in GSRCC. This subpopulation exhibited marked genomic instability with significant CNV accumulation (Figure 6A,B) and a more terminally differentiated state, as indicated by lower CytoTRACE scores (Figure 6C–E). These characteristics were consistently validated in an independent cohort, where MUCL3-high cells similarly showed lower CytoTRACE scores and elevated CNVs (Supplementary Figure S7A–D). To further validate this differentiation trajectory, we performed pseudotime analysis, which placed MUCL3^+^ Cluster 1 at the terminal end (Supplementary Figure S8). Hallmark pathway analysis revealed broad activation of pro-tumorigenic processes in this subpopulation, including inflammatory responses (TNFA/NFKB, IL2-STAT5, Interferon-gamma), pro-proliferative signaling (KRAS, mTORC1), metabolic reprogramming, and key processes driving invasion (Epithelial–Mesenchymal Transition, TGF-Beta signaling). (Figure 6F). Hallmark enrichment analysis using MUCL3-high versus MUCL3-low cells in the validation cohort confirmed consistent enrichment of EMT, inflammatory response, and KRAS signaling pathways (Supplementary Figure S9). Concordantly, gene set variation analysis showed elevated metastasis- and angiogenesis-related scores (Figure 6G), suggesting potential association with invasive and pro-angiogenic capabilities relevant to peritoneal dissemination. Within this subpopulation, we further investigated the co-upregulated gene *TFF1*. Single-cell correlation analysis in the validation cohort revealed that *TFF1* expression correlated positively with *MUCL3* and was significantly enriched in MUCL3^+^ GSRCC cells (Supplementary Figure S10). qPCR confirmed *TFF1* upregulation in GSRCC tissues compared to paracancerous and AC tissues (Figure 6H). Functional validation in gastric cancer lines (MGC803, MKN45) indicated that recombinant TFF1 (2 μg·mL^−1^, 24 h) was associated with increased migration in Transwell assays (Figure 6I,J). These findings suggest that TFF1 may serve as a candidate effector contributing to the aggressive phenotype associated with the MUCL3^+^ molecular signature.

## 4. Discussion

The clinical management of advanced AC remains a formidable challenge, particularly for GSRCC [30,31,32], which is hampered by its distinct pathobiology and consequent resistance to conventional therapies, which frequently leads to chemotherapy failure and metastasis [33,34,35,36,37]. While certain regimens can slow disease progression [38], they do not yet represent personalized therapies capable of achieving optimal outcomes. Here, we provide protein-level validation of MUCL3 as a specific marker of signet-ring cells and reveal that this subpopulation exhibits metastasis-associated molecular features and elevated *TFF1* expression. In contrast to these broadly expressed or heterogeneously expressed markers, *MUCL3* exhibits a restricted expression pattern specifically enriched in SRCC cells. Unlike *MUC1*, which is widely expressed across various gastric cancer subtypes and associated with poor prognosis, MUCL3 expression in GSRCC is tightly linked to malignant transformation and aggressive features. While *CDH1* loss, *KRAS* mutations, and *Claudin18.2* expression have been implicated in SRCC pathogenesis [39,40,41,42], their heterogeneous expression or mutational status limits their utility as reliable subpopulation-specific biomarkers.

To dissect the cellular architecture of GSRCC, we began with a systematic analysis of gastric epithelial cells, which revealed marked inter-patient heterogeneity. Focusing subsequently on GSRCC, we uncovered a fundamental restructuring of the epithelium. This restructuring was characterized not only by the loss of functional lineages (as reported during SPEM transition [43]) but, more significantly, by a pronounced expansion of the mucinous Mucous_muc5ac subpopulation. These combined findings reveal an extreme, mucin-biased reprogramming of differentiation. To molecularly define this reprogrammed epithelium, we identified a GSRCC-upregulated gene signature encompassing both prognostic genes (e.g., *GAST*, *NEAT1*) and mucus-related factors (e.g., *MUC5AC*, *TFF1*, *TFF2*). The mucus-related molecules were specifically enriched in the Mucous_muc5ac subpopulation at single-cell resolution. More importantly, immunofluorescence first confirmed their protein-level co-expression in morphologically defined signet-ring cells, solidifying this subpopulation as the principal source of GSRCC-defining molecules. This finding clearly distinguishes our identified factors *TFF1* and *TFF2* from the previously studied thyroid transcription factors *TTF1* and *TTF2* [20]. Furthermore, although *MUC5AC* and *TFF1* were significantly upregulated in GSRCC, neither represents a tumor-specific marker, as both are normally expressed in gastric pit epithelial cells. Their overexpression in GSRCC likely reflects the hijacking of normal gastric pit differentiation programs rather than de novo oncogenic activation.

Having characterized the expanded Mucous_muc5ac subpopulation, we next examined its malignant features. This study revealed that the Mucous_muc5ac subpopulation in GSRCC is characterized by a malignant phenotype combining genomic instability (increased CNVs) and a dedifferentiated state (low CytoTRACE scores). From this subpopulation, we isolated a GSRCC-specific MUCL3-high subset and validated MUCL3 protein expression in signet-ring cells, defining it as a novel diagnostic marker. Recent studies have established *MUCL3* as a key NF-κB-driven gene upregulated in H. pylori-positive GC, serving as a marker of poor prognosis and a potential therapeutic target [44]. Its role extends to the tumor microenvironment, where *MUCL3* is enriched in pro-angiogenic cancer-associated fibroblast subpopulations, suggesting a function in promoting angiogenesis [45]. Furthermore, in pancreatic ductal AC, *MUCL3* is identified as a characteristic driver of the classical subtype and is associated with poor outcomes, potentially by regulating mucin glycosylation and cell junctions [46]. Notably, in the stomach, *MUCL3* is a novel lineage marker upregulated during the transdifferentiation of parietal cells to Spasmolytic polypeptide-expressing metaplasia (SPEM), a key precancerous lesion [47]. These findings highlight MUCL3’s multifaceted role in tumor progression and stromal interaction, positioning it as a compelling candidate for future investigation in GSRCC.

To functionally delineate the MUCL3^+^ subpopulation, we performed an integrated multi-omics analysis to characterize its biological properties. Notably, although the overall Mucous_muc5ac subpopulation exhibited a high CytoTRACE score (indicative of a less differentiated, progenitor-like state), the MUCL3^+^ subpopulation displayed a comparatively lower CytoTRACE score. This suggests that the MUCL3^+^ subpopulation may have originated from the differentiation of other cells within the parental population that possess stronger progenitor-like characteristics, reflecting functional heterogeneity within this cell group. In contrast to the signet-ring subclusters identified from epithelial cells by Zhao et al. [20], the MUCL3^+^ subpopulation identified here from the MUC5AC^+^ mucous subpopulation demonstrated significant genomic instability (high CNV score) and a distinct dedifferentiated phenotype. Pathway enrichment analysis revealed that this subpopulation co-opts a coordinated network of tumor-promoting pathways. This network is characterized by pro-invasive programs (TGF-β signaling and epithelial–mesenchymal transition), a potent inflammatory-immune milieu (TNF-α/NF-κB signaling, interferon-γ response, and inflammatory response), and robust proliferative drive (mTORC1 signaling). The concerted activation of these modules likely underlies the subpopulation’s aggressive phenotype. Particularly striking is the markedly elevated enrichment of gene sets linked to “metastasis” and “angiogenesis” in this subpopulation, offering a plausible cellular basis for the pronounced tendency of GSRCC to undergo peritoneal implantation and lymphatic dissemination.

Within this subpopulation, we further investigated the co-upregulated gene *TFF1*. qPCR analysis revealed that *TFF1* expression was significantly elevated in GSRCC tissues compared to both AC tissues and matched adjacent normal tissues. While *TFF1* has been implicated as a pro-oncogenic factor in multiple cancers [48,49,50], its overexpression-driven role in GSRCC remains largely unknown. In the present study, the marked upregulation of *TFF1* observed specifically within the MUCL3^+^ signet-ring cell subpopulation suggests that *TFF1* may contribute to the malignant phenotype of this subpopulation. This notion was further supported by in vitro functional experiments, which indicated that exogenous TFF1 protein was associated with increased migratory capacity of gastric cancer cells.

Based on the molecular characteristics outlined above, we propose a potential mechanism for the malignant progression of GSRCC: tumor cells hijack the normal differentiation program of gastric pit epithelial cells (particularly the mucin secretory pathway), thereby acquiring genomic instability, a dedifferentiated state, enhanced metastatic potential, robust angiogenic activity, and activation of multiple pro-oncogenic pathways, collectively driving the aggressive advancement of the tumor. Several notable features of this study include: (1) expansion of a MUC5AC^+^ mucous subpopulation in GSRCC; (2) identification of a MUCL3^+^ signet-ring cell subset with high *TFF1* expression; (3) multi-omics characterization of its metastasis-associated pathways; and (4) validation of TFF1 as a candidate effector. This study has several limitations as well. First, the discovery cohort sample size is relatively limited, though validation using independent public datasets supports generalizability. Due to the limited number of GSRCC cases (*n* = 7) available for immunohistochemical validation, case-level sensitivity and specificity of *MUCL3* as a diagnostic marker remain to be determined. Second, *MUCL3* expression strongly correlates with aggressive molecular features, but causality is unestablished; thus, evidence for metastatic potential is molecular and requires direct in vivo demonstration (e.g., tail vein injection) via future knockdown/overexpression experiments. Third, TFF1 validation was performed in conventional cell lines rather than signet-ring lines; future studies using MUCL3^+^ GSRCC models (e.g., NUGC4 or organoids) are required to confirm TFF1’s role, and the precise mechanisms remain to be elucidated.

## 5. Conclusions

This study identified a distinct subpopulation of signet-ring cells specifically overexpressing *MUCL3* in GSRCC by integrating single-cell transcriptomics with multi-omics approaches. This subpopulation was not only identified as the signet-ring cells in GSRCC for the first time, but also showed malignant phenotypes including genomic instability, dedifferentiation, metastasis-associated features, and pro-angiogenic activity. Functional validation further identified TFF1 as a candidate effector within this subpopulation that is associated with gastric cancer cell migration, warranting further investigation for GSRCC metastasis. Our findings provide a novel theoretical foundation and experimental basis for molecular diagnosis and further mechanistic investigation of GSRCC.

## Data Availability

The raw scRNA-seq data have been deposited in the GSA-Human database under accession number HRA017882. The data are currently under controlled access and are available upon reasonable request.

## Supplementary Materials

The following supporting information can be downloaded at: https://www.mdpi.com/article/10.3390/cells15100857/s1, Figure S1: Cohort characteristics and single-cell data sources; Figure S2: Cellular landscape of the validation cohort; Figure S3: Identification and quantification of the Mucous_muc5ac epithelial subpopulation in the validation cohort; Figure S4: Molecular characterization and prognostic gene analysis of epithelial cells in the validation cohort; Figure S5: Prognostic impact of genes upregulated in the GSRCC Mucous_muc5ac subpopulation; Figure S6: Analysis of MUCL3 expression in the validation cohort: overall epithelial cell levels and distribution across the reclustered Mucous_muc5ac subpopulation; Figure S7: Molecular characterization of the Mucous_muc5ac subclusters in the validation cohort; Figure S8: Pseudotime analysis reveals differentiation trajectory among the four subclusters of Mucous_muc5ac; Figure S9: Hallmark pathway enrichment analysis of MUCL3-high vs MUCL3-low populations; Figure S10: TFF1 is enriched in MUCL3^+^ GSRCC cells at single-cell resolution; Table S1: Distribution of cell numbers across Mucous_muc5ac subclusters by patient in the discovery cohort.

## Abbreviations

The following abbreviations are used in this manuscript:

GC	Gastric Cancer
GSRCC	Gastric signet-ring cell carcinoma
AC	Adenocarcinoma
scRNA-seq	Single-cell RNA sequencing
MUCL3^+^	Mucin-Like 3-positive
DEG	Differentially Expressed Gene
mIF	Multiplex immunofluorescence
