# Supplementary material for: Single-Cell Transcriptomic Profiling Uncovers a Metastasis-Associated MUCL3+ Signet-Ring Cell Subpopulation in Gastric Cancer
Source: Cells. 2026 May 8;15(10):857. doi: 10.3390/cells15100857 (PMC13204890; doi:10.3390/cells15100857)
Supplement: Supplementary file 1 [file cells-15-00857-s001.zip › cells-4238189-supplementary.pdf]

Supplementary Figures

Supplementary Figure1 | Cohort characteristics and single-cell data sources.

Supplementary Figure 2 | Cellular landscape of the validation cohort.

Supplementary Figure 3 | Identification and quantification of the Mucous_muc5ac epithelial subpopulation in the validation cohort.

Supplementary Figure 4 | Molecular characterization and prognostic gene analysis of epithelial cells in the validation cohort

Supplementary Figure5 | Prognostic impact of genes upregulated in the GSRCC Mucous_muc5ac subpopulation.

Supplementary Figure 6 | Analysis of MUCL3 Expression in the Validation Cohort: Overall Epithelial Cell Levels and Distribution across the Reclustered Mucous_muc5ac Subpopulation

Supplementary Figure 7 | Molecular characterization of the Mucous_muc5ac subclusters in the validation cohort.

Supplementary Figure 8| Pseudotime analysis reveals differentiation trajectory among the four subclusters of Mucous_muc5ac.

Supplementary Figure 9| Hallmark pathway enrichment analysis of MUCL3-high vs MUCL3-low populations

Supplementary Figure S10. TFF1 is enriched in MUCL3^{+} GSRCC cells at single-cell resolution.

Supplementary Table S1. Distribution of cell numbers across Mucous_muc5ac subclusters by patient in the discovery cohort

A

PATIENT	GENDER	AGE	TNM	CARCINOMA TYPE	AFFECTED AREA	lymphatic transfer
SRCC1	Female	77	T1N0M0	Gastric signet ring cell cancer	Lesser Curvature	NO
ACT1	Male	66	T4N0M0	Poorly Differentiated	Gastric Body	NO
ACT2	Female	36	T2N0M0	Poorly Differentiated	Cardia	NO
SRCC2	Female	66	T3N1M0	Poorly differentiated with gastric signet ring cell cancer	Gastric Body	YES
SRCC3	Male	49	T3N3M0	Poorly differentiated with gastric signet ring cell cancer	Gastric Body	YES

B

Sample Name	Individual ID	CARCINOMA TYPE
ncACT_159	HRI322223	Moderately differentiated gastric cancer tissue
ncACT_160	HRI322224	Moderately differentiated gastric cancer tissue
ncACT_161	HRI322225	Moderately differentiated gastric cancer tissue
ncACT_163	HRI322227	Poorly differentiated gastric cancer tissue
ncACN_172	HRI322236	Paracancerous tissue of moderately differentiated gastric cancer
ncACN_173	HRI322237	Paracancerous tissue of poorly differentiated gastric cancer
ncSRCCT_165	HRI322229	Poorly differentiated with gastric signet ring cell cancer
ncSRCCT_166	HRI322230	Poorly differentiated with gastric signet ring cell cancer
ncSRCCT_167	HRI322231	Poorly differentiated with gastric signet ring cell cancer
ncSRCCT_168	HRI322232	Gastric signet ring cell cancer
ncSRCCT_169	HRI322233	Gastric signet ring cell cancer
ncSRCCT_170	HRI322234	Gastric signet ring cell cancer
ncSRCCT_171	HRI322235	Gastric signet ring cell cancer

Supplementary Figure 1 | Cohort characteristics and single-cell data sources.

(A) Detailed clinical and pathological information for patients in the experimental discovery cohort.

(B) Detailed clinical and pathological information for patients in the independent validation cohort.

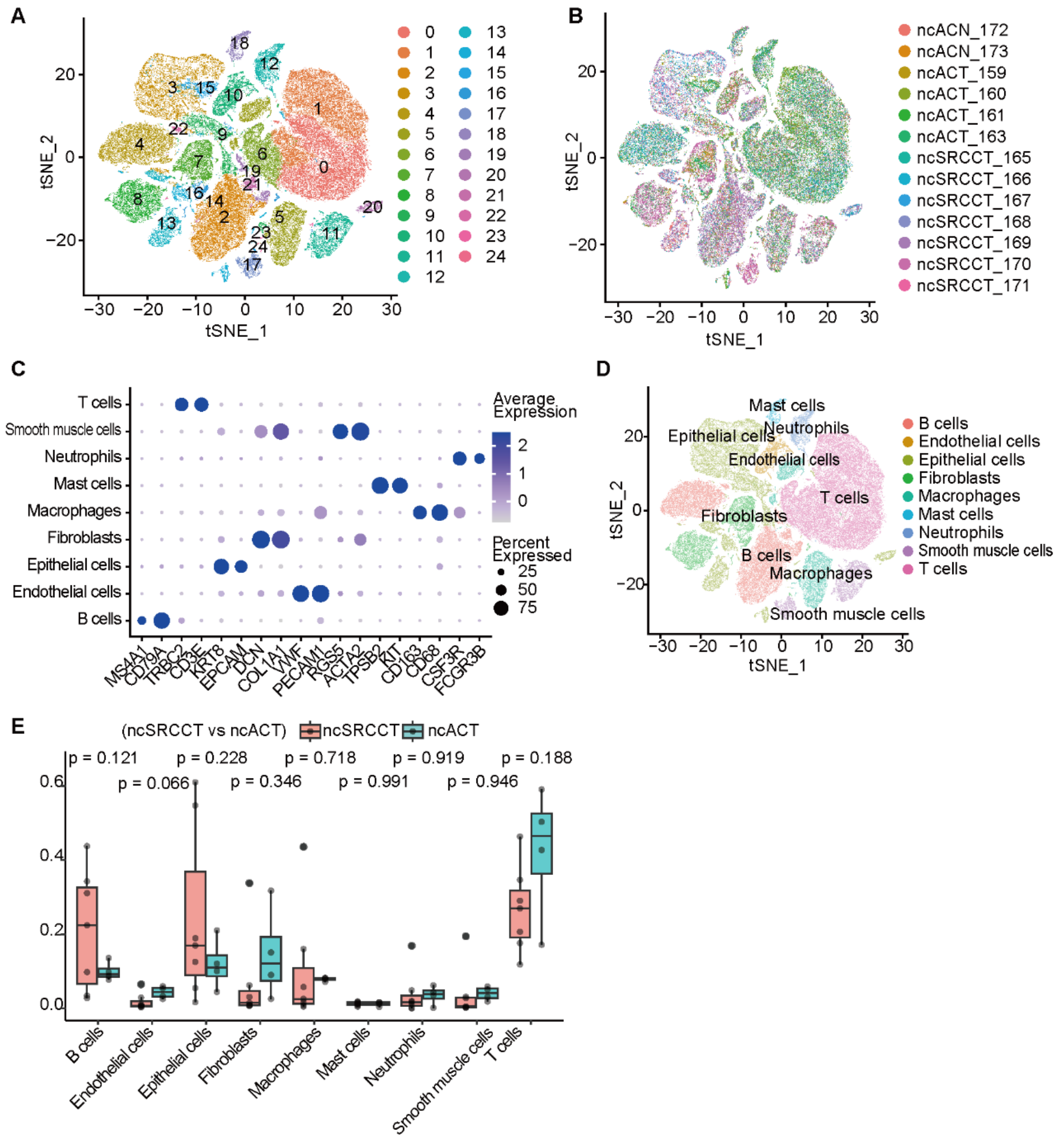

Supplementary Figure 2 | Cellular landscape of the validation cohort.

(A) t-SNE plot displaying 25 unsupervised clusters identified from all cells in the validation cohort.

(B) t-SNE plot colored by sample source.

(C) Dot plot illustrating the expression of canonical marker genes used to annotate the nine major cell types.

(D) t-SNE visualization of cells, colored by their annotated cell type identity.

(E) Bar graph comparing the average proportional composition of major cell types between GSRCC and AC tissues.

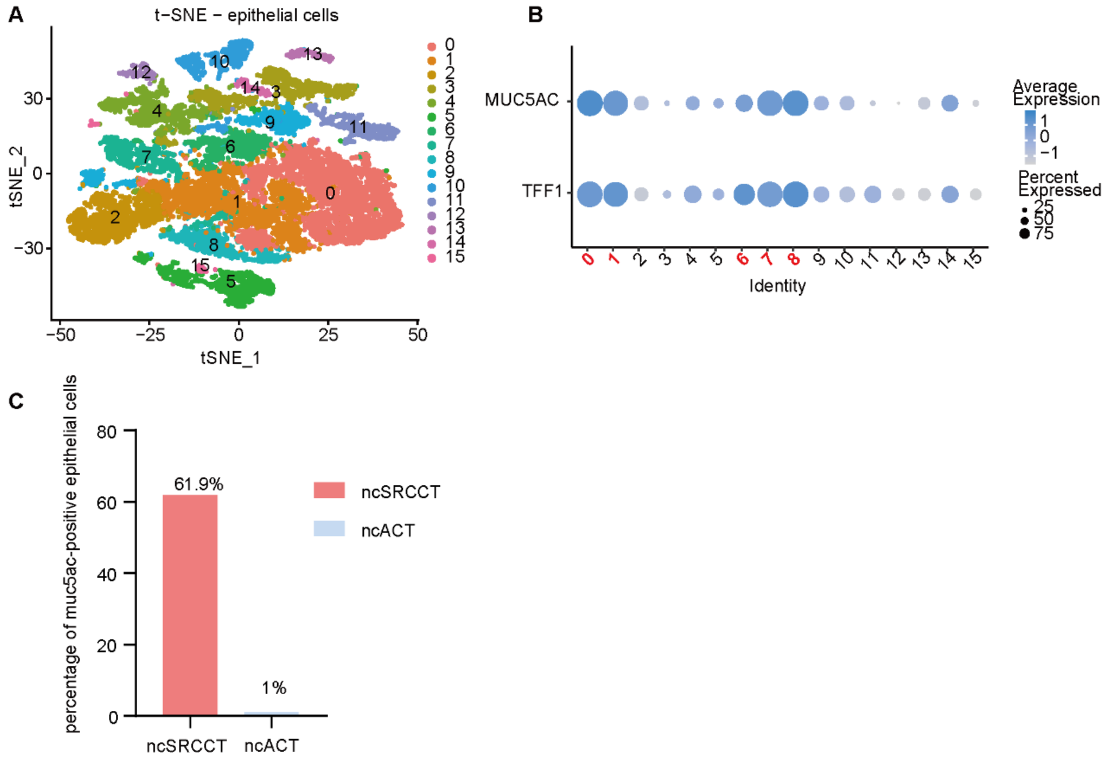

Supplementary Figure 3 | Identification and quantification of the Mucous_muc5ac epithelial subpopulation in the validation cohort.

(A) t-SNE plot showing the clustering of epithelial cells into distinct subpopulations.

(B) Dot plot of marker gene expression across epithelial subclusters. Clusters 0, 1, 6, 7, and 8, which co-express TFF1 and MUC5AC, were annotated as the Mucous_muc5ac subpopulation.

(C) Box plot comparing the relative abundance of the Mucous_muc5ac subpopulation within the epithelial compartment of GSRCC versus AC tissues.

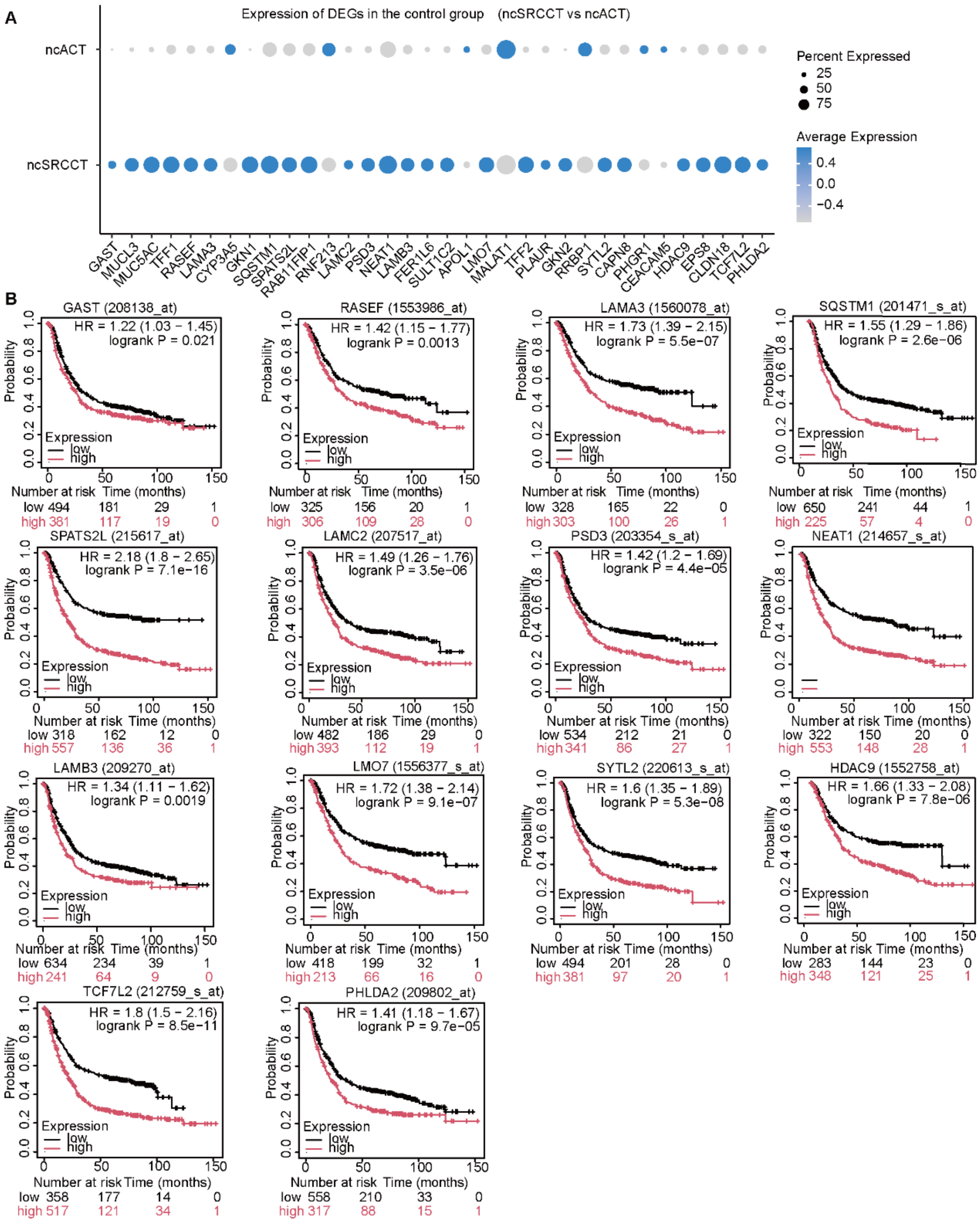

Supplementary Figure 4 | Molecular characterization and prognostic gene analysis of epithelial cells in the validation cohort

(A) Dot plot displaying differential expression of target genes (e.g., MUC5AC, TFF1, MUCL3) in epithelial cells between GSRCC and AC tissues within the validation cohort.

(B) Kaplan–Meier

survival curves illustrating the association between expression levels of genes significantly correlated with poor prognosis in gastric cancer patients and overall survival (OS).

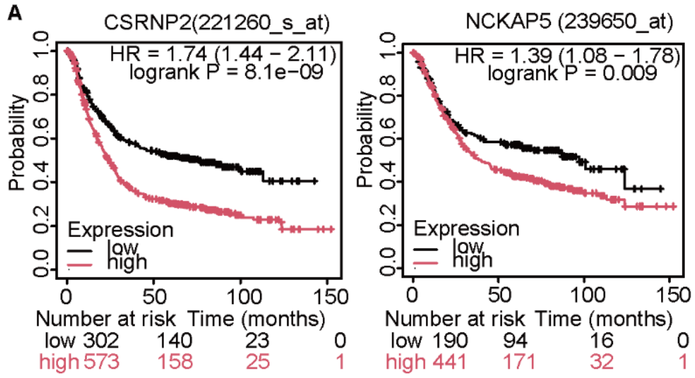

Supplementary Figure 5 | Prognostic impact of genes upregulated in the GSRCC Mucous_muc5ac subpopulation.

（A）Kaplan-Meier survival analysis showing the association between high expression of the upregulated DEGs and patient overall survival.

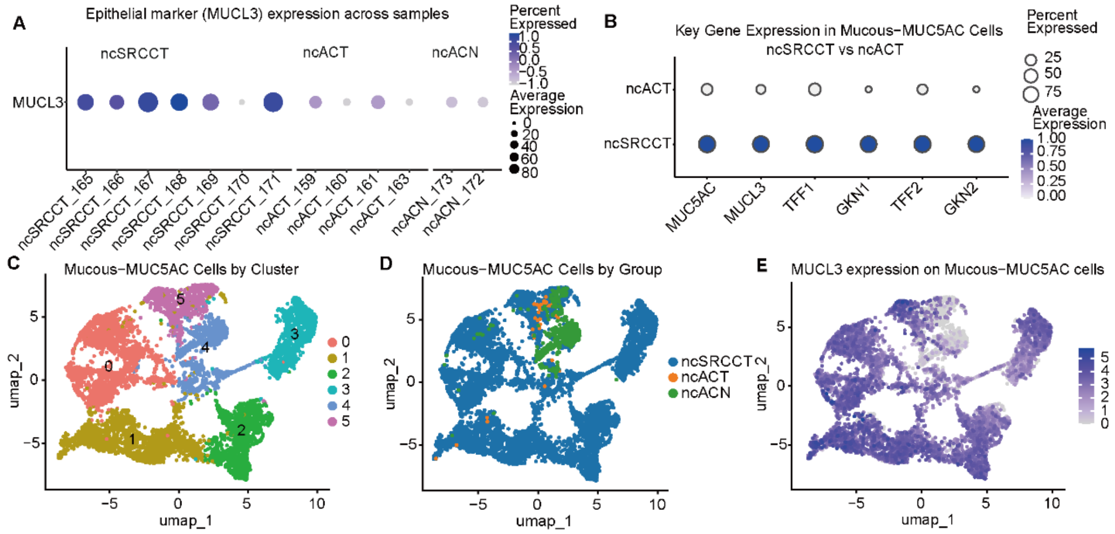

Supplementary Figure 6 | Analysis of MUCL3 Expression in the Validation Cohort: Overall Epithelial Cell Levels and Distribution across the Reclustered Mucous_muc5ac Subpopulation

(A) Dot plot showing the expression level of MUCL3 in epithelial cells across all samples of the validation cohort.

(B) Dot plot displaying the differential expression of target genes (including MUC5AC, TFF1, and MUCL3) in epithelial cells between GRCC and AC tissues within the validation cohort.

(C) UMAP plot of Mucous_muc5ac cells colored by cluster.

(D) UMAP plots of Mucous_muc5ac cells categorized by tissue origin.

(E) UMAP visualization of MUCL3 expression distribution in the Mucous_muc5ac subpopulation; the color gradient represents expression levels from low (light) to high (dark).

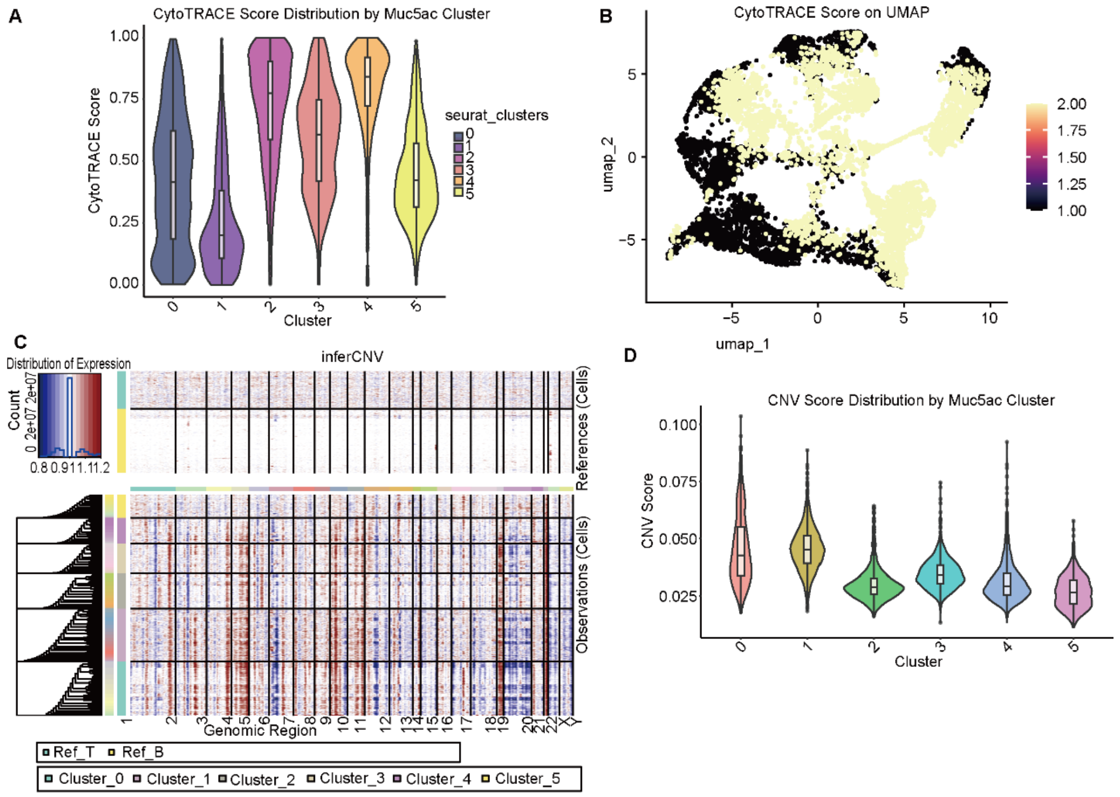

Supplementary Figure 7 | Molecular characterization of the Mucous_muc5ac subclusters in the validation cohort.

(A) Violin plots comparing the differentiation potential (CytoTRACE scores) across Mucous_muc5ac subclusters.

(B) UMAP visualization of CytoTRACE scores projected onto the Mucous_muc5ac subpopulation.

(C) Heatmap of inferCNV-predicted copy number variations (CNVs) across Mucous_muc5ac subclusters.

(D) Violin plots displaying the distribution of CNV scores for each Mucous_muc5ac subcluster.

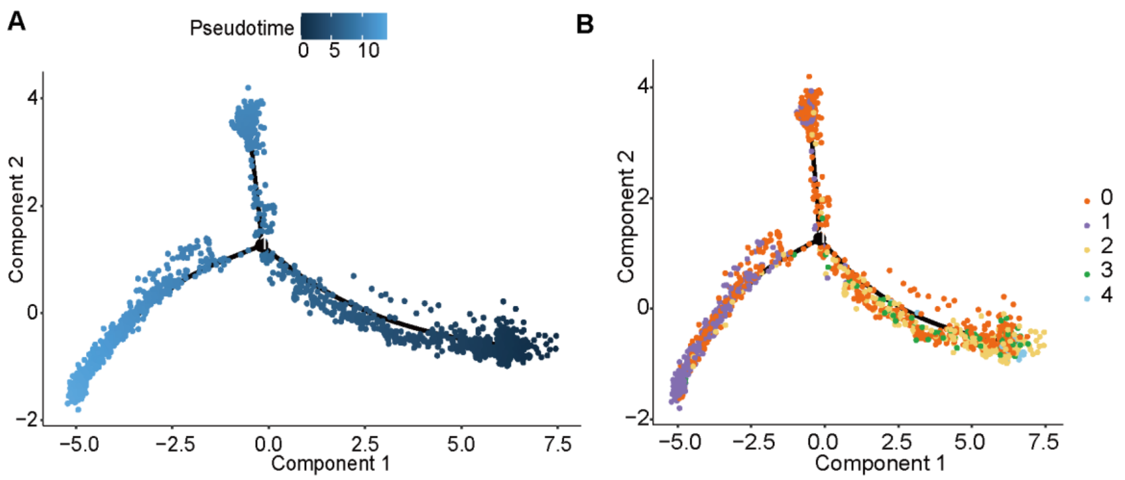

Supplementary Figure8| Pseudotime analysis reveals differentiation trajectory among the four subclusters of Mucous_muc5ac.

(A)Cells colored by pseudotime. Smaller pseudotime values (dark blue) indicate less differentiated states, while larger values (light color) indicate more differentiated states. (B) Cells colored by subcluster identity. Each color represents one of the four subclusters, showing their distribution along the differentiation trajectory.

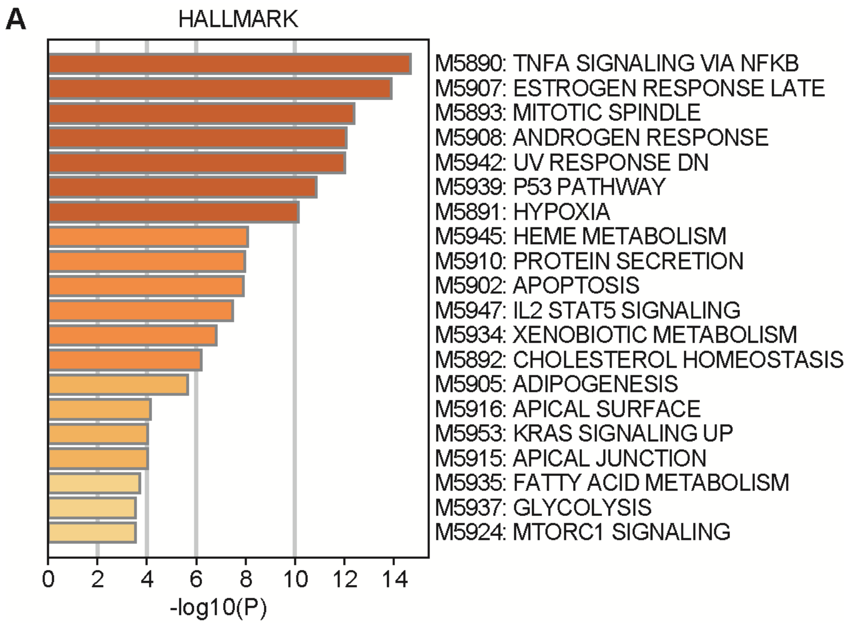

Supplementary Figure 9| Hallmark pathway enrichment analysis of MUCL3-high vs MUCL3-low populations

(A)MUC5AC-positive cells were stratified into MUCL3-high (top 25%) and MUCL3-low (bottom 25%) groups. The top 1000 upregulated genes (|logFC|≥0.25, FDR < 0.05) were analyzed using Metascape (http://metascape.org) with default parameters (FDR < 0.05, min overlap≥ 3). Bar plots show the top 20 enriched pathways in discovery cohort.

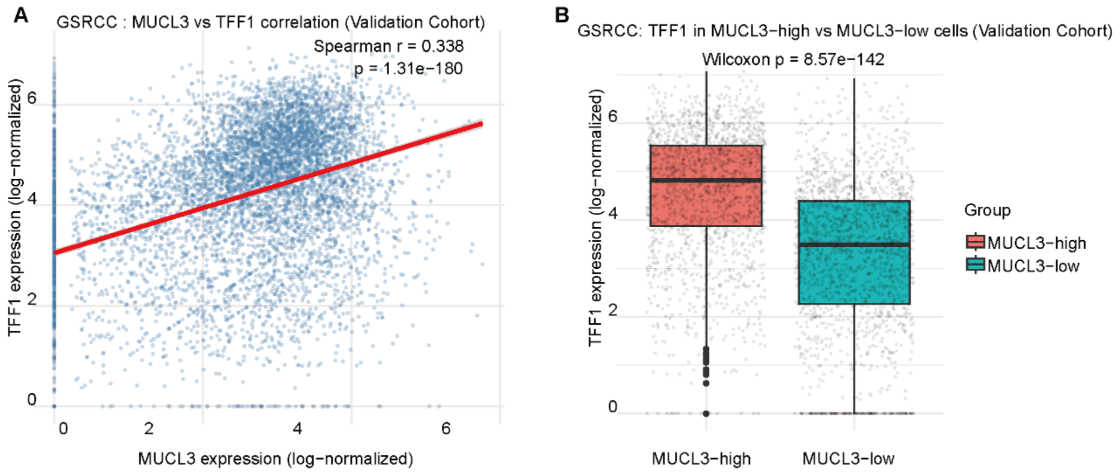

Supplementary Figure 10| TFF1 is enriched in MUCL3⁺ GSRCC cells at single-cell resolution.

(A) Scatter plot showing the correlation between MUCL3 and TFF1 expression in GSRCC cells from the validation cohort (n = 6,754). Red line indicates linear regression fit with 95% confidence interval. Spearman's ρ = 0.338, $p < 2.2e-16$.

(B) Boxplot comparing TFF1 expression between MUCL3-high (top 25%) and MUCL3-low (bottom 25%) GSRCC cells. MUCL3-high cells show significantly higher TFF1 expression (Wilcoxon test, $p < 2.2e-16$).

Supplementary Table S1.

Distribution of cell numbers across Mucous_muc5ac subclusters by patient in the discovery cohort

Sample	cluster0	Cluster1	Cluster2	Cluster3	Cluster4
SRCCT1	165	30	21	2	0
SRCCT2	172	229	20	3	0
SRCCT3	104	216	27	13	0
ACT1	62	1	74	5	0
ACT2	127	0	29	21	3

Legend: Per-patient cell counts for each of the five Mucous_muc5ac subclusters (Clusters 0–4) in the discovery cohort. Cluster 1, the GSRCC-enriched subcluster, contained 30, 229, and 216 cells from the three GSRCC patients, respectively, demonstrating consistent enrichment across independent patients. In contrast, Cluster 1 was nearly absent in the two AC patients (1 and 0 cells).
